# Within-subject reproducibility varies in multi-modal, longitudinal brain networks

**DOI:** 10.1038/s41598-023-33441-3

**Published:** 2023-04-24

**Authors:** Johan Nakuci, Nick Wasylyshyn, Matthew Cieslak, James C. Elliott, Kanika Bansal, Barry Giesbrecht, Scott T. Grafton, Jean M. Vettel, Javier O. Garcia, Sarah F. Muldoon

**Affiliations:** 1 Neuroscience Program, University at Buffalo, SUNY, Buffalo, NY 14260 USA; 2 School of Psychology, Georgia Institute of Technology, Atlanta, GA 14260 USA; 3 U.S. CCDC Army Research Laboratory, Aberdeen Proving Ground, Aberdeen, MD 21005 USA; 4 Department of Bioengineering, University of Pennsylvania, Philadelphia, PA 19104 USA; 5 Department of Psychological and Brain Sciences, University of California, Santa Barbara, CA 93106 USA; 6 Department of Biomedical Engineering, Columbia University, New York, NY 10027 USA; 7 Institute for Collaborative Biotechnologies, University of California, Santa Barbara, CA 93106 USA; 8 Department of Mathematics and CDSE Program, University at Buffalo, SUNY, Buffalo, NY 14260 USA

**Keywords:** Network models, Imaging, Network topology

## Abstract

Network neuroscience provides important insights into brain function by analyzing complex networks constructed from diffusion Magnetic Resonance Imaging (dMRI), functional MRI (fMRI) and Electro/Magnetoencephalography (E/MEG) data. However, in order to ensure that results are reproducible, we need a better understanding of within- and between-subject variability over long periods of time. Here, we analyze a longitudinal, 8 session, multi-modal (dMRI, and simultaneous EEG-fMRI), and multiple task imaging data set. We first confirm that across all modalities, within-subject reproducibility is higher than between-subject reproducibility. We see high variability in the reproducibility of individual connections, but observe that in EEG-derived networks, during both rest and task, alpha-band connectivity is consistently more reproducible than connectivity in other frequency bands. Structural networks show a higher reliability than functional networks across network statistics, but synchronizability and eigenvector centrality are consistently less reliable than other network measures across all modalities. Finally, we find that structural dMRI networks outperform functional networks in their ability to identify individuals using a fingerprinting analysis. Our results highlight that functional networks likely reflect state-dependent variability not present in structural networks, and that the type of analysis should depend on whether or not one wants to take into account state-dependent fluctuations in connectivity.

## Introduction

The introduction of network theory to neuroscience has increased our understanding of the brain’s functional and structural organization. This powerful tool has given new insights into how higher order brain functions arise^1,2^ and how changes can lead to pathology^3^. However, questions have been raised regarding the reliability of brain network properties given the effects of noise in the signal, particularly in fMRI^4–6^. Still, despite the presence of noise, brain networks have been found to exhibit consistent properties over time among individual network connections and in higher order properties, such as the clustering coefficient, characteristic path length, and assortativity, for structural connectivity as measured with dMRI^7–11^, fMRI^12–25^ and EEG/MEG^18,26,27^. Unfortunately, most studies thus far have been limited to the analysis of a single imaging modality and/or few scanning sessions, raising questions about how reliable these properties are over longer times and across modalities.

While it is clear that there is some level of reliability in network properties within an individual over time, it is also important to understand how the state of the brain (e.g., resting wakefulness versus active task situations^28^), and the neural methodology (e.g., fMRI versus EEG) contributes to this reliability across multiple days. The “resting” brain (e.g., default mode network) is a state that has been shown to be metabolically demanding^29^ and associated not only with task performance (e.g., Ref.^30^) but also disease (e.g., Ref.^31^), very much similar to task-related activity; however, the “resting” brain is fundamentally different from task-related activity, as engagement in a task requires precise recruitment of and coordination between regions of the brain^28^. Also, in a field with a variety of diverse methodologies (e.g., fMRI, EEG, MEG, PET, etc.), neuroscience researchers draw conclusions from methods that are measuring fundamentally different neural properties. For example, fMRI is an indirect measurement of neural activity, as it measures oxygenation and neural activity is inferred. In contrast, EEG, a “direct” measurement, is measured on the scalp and filtered by a variety of tissues and bone separating the scalp from the brain. In terms of reliability, experimental design and task demands have shown to contribute to reliability in fMRI^32,33^, and EEG suffers from a large variety of factors that could impact reliability as well^34^. However, the reliability of networks derived from simulatneous fMRI and EEG has not been extensively studied and comparisons has been limited to resting-state^35^.

In addition to studying reliability within an individual over time, one can also ask about how network properties differ between individuals. Indeed, recent work has shown that brain networks can provide insight into the unique features associated with a person^19,36–38^. A giant leap toward the goal of understanding differences in brain networks was made with the finding that functional brain activity has unique features that can identify a person in a group, similar to a fingerprint^39^. This fingerprinting property has also been found in structural connectomes^40,41^. Fingerprinting is important because it allows neuroimaging analyses to focus on the individual and not only on group-level differences^39^.

To further understand reliability in brain networks over time, across different states, and across modalities, we quantified within- and between-subject reliability in a rich longitudinal and multi-modal dataset consisting of dMRI and simultaneous EEG-fMRI recording during resting-state and multiple tasks. Comparing reliability across different imaging modalities is particularly important because in recent years, studies have fused multiple modalities to address outstanding questions in neuroscience. Further, while previous work has shown that diffusion MRI is highly reliable with sufficient images^42^, other work has shown that structural changes can occur over a short time period with exercise^43^, working memory training^44^, and changes in sleep patterns^45^. Understanding the relationship between structure–function coupling and reliability over time and/or across tasks can therefore aide in studies that incorporate analysis across modalities.

Importantly, the data set studied here was part of a larger study examining naturalistic sleep variability in individuals^46^. Here, we do not focus on the effects of variation in sleep pressure (i.e., homeostatic sleep drive or the need to sleep), but instead note that due to the study design, subjects varied in the amount of sleep pressure they experienced during each imaging session, presumably augmenting variability within- and between-subjects’ functional brain network over time. We examine both structural and functional brain networks in this data set to study reliability of individual connections and higher order network statistics. To create structural networks, dMRI imaging was used to perform tractography and network connections were defined as the density of streamlines between brain regions. fMRI networks were constructed using the Pearson-Product Correlation to quantify the magnitude of the statistical relationship in the BOLD signal between brain regions. For EEG, the time-series signal from each sensor was first separated into traditional frequency bands of δ (1–3 Hz), θ (4–7 Hz), α (8–13 Hz), β (14–30 Hz) and γ (30–60 Hz), and functional connectivity was calculated using the debiased-weighted Phase-Lag Index (dwPLI) which quantifies phase synchronization between sensors based on the consistency of the lag between the instantaneous phases of two sensors^47^.

In the current work we evaluate: (1) which brain connections and network measures are most reliable within- and between-individuals; (2) how reliability varies across state and modality; and (3) how the different imaging modalities, dMRI, fMRI, and EEG, perform in a fingerprinting analysis to identify an individual.

## Results

We analyzed the reproducibility of brain network properties derived from structural and functional brain imaging using the intra-class correlation (*ICC*). For the dMRI analysis, this involved analyzing brain networks from 25 subjects across 8 sessions for a total of 200 structural networks. For the fMRI and EEG analysis, a tradeoff between maximizing subjects and sessions was made across resting-state and tasks resulting in a range from 17 to 26 subjects, each with 6 sessions (see “Materials and methods” section for details). For each connection in the network or network measure, the ICC values were calculated using the mean square values for the subject and session terms.

### Reliability of individual connections

**Figure 1:**
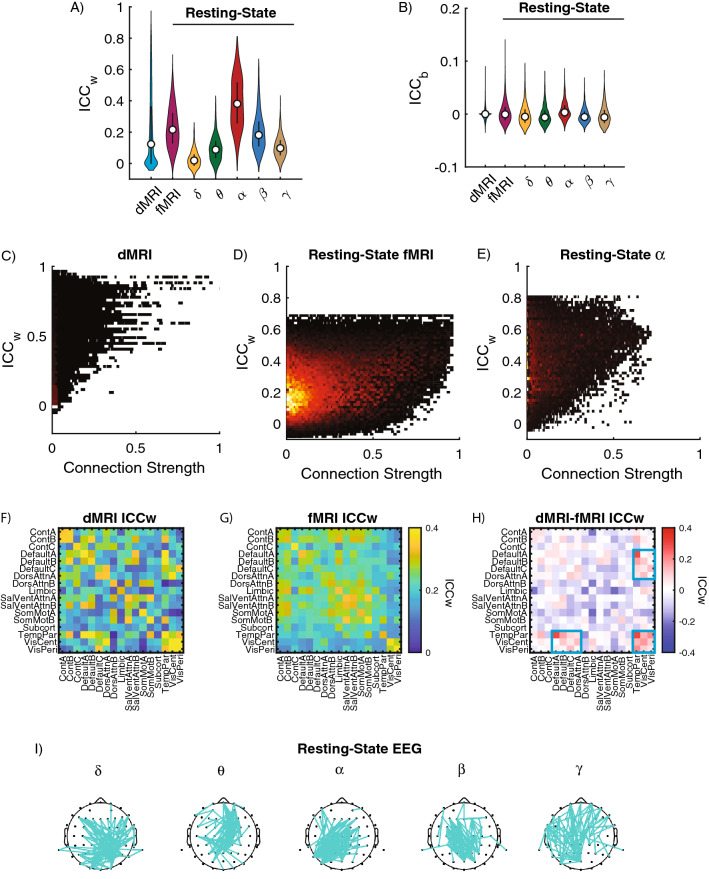
Reliability of individual connections. (**A**) Distribution of ICC_w_ and (**B**) ICC_b_ for dMRI and resting-state fMRI and EEG frequency bands. For each violin plot, the central dot indicates the median, and the line indicates the 25th–75th percentiles. (**C**–**E**) Distribution plots showing the connection strength of ICC_w_ scores for (**C**) dMRI; (**D**) Resting-State fMRI and (**E**) EEG-α. (**F**–**H**) Average reliability of connection within and between cognitive systems for (**F**) dMRI and (**G**) resting-state fMRI. (**H**) Differences in average ICC_w_ scores across cognitive systems between dMRI minus the fMRI. Blue boxes highlight connections between cognitive systems in which reliability was stronger in dMRI. (**I**) Connections with ICC_w_ scores in top 5% for δ, θ, α, β and γ frequency bands plotted on the scalp for resting-state EEG. Cognitive systems are defined as Cont: Control A/B/C, Default: Default Mode A/B/C, DorsAttn: Dorsal Attention A/B, Limbic, SalVentAtt: Salience/Ventral Attention A/B, SomMot: Somatomotor A/B, Subcortical, TempPar: Temporal Parietal, VisCent: Visual Central, VisPer: Visual Peripheral.

We first assessed the reliability of individual connections between brain regions or sensors. We calculated the ICC within a subject (ICC_w_) and between subjects (ICC_b_) for each connection across the three imaging modalities. As expected, we found that across imaging modalities, individual network connections are more reliable within- than between-subjects (Fig. 1A,B). Across imaging modalities, individual edges exhibit high variability in their reliability scores, with ICC_w_ values ranging from poor (< 0.4) to excellent (> 0.8) reliability (Fig. 1A). By contrast, ICC_b_ scores had consistently poor (< 0.2) reliability across all imaging modalities (Fig. 1B). For dMRI, the mean ICC_w_ was 0.21 ± 0.24 (SD) and the mean ICC_b_ score was -1 × 10^–4^ ± 0.01 (SD). For resting-state fMRI the mean ICC_w_ was 0.23 ± 0.13(SD). Lastly, for the EEG the α-band had the highest mean ICC_w_ (0.39 ± 0.16(SD) compared to the other frequencies [δ: 0.03 ± 0.05(SD); θ: 0.09 ± 0.08(SD); β: 0.20 ± 0.12(SD); γ: 0.10 ± 0.07(SD)]. To assess differences across imaging modalities, we used a one-way ANOVA where dMRI, fMRI, and each EEG frequency band (δ, θ, α, β, γ) were treated as sperate groups, and we found significant differences in the ICC_w_ (F_7,85249_ = 2241; p_corrected_ <  < 0.001; η^2^ = 0.18). One important feature is the long-tail distribution in the dMRI ICC_w_ indicating that a small number of connections have excellent (> 0.8) reliability. We additionally looked to see if there was a relationship between connection strength and reliability (Fig. 1C–E).

We next assessed if for dMRI and resting-state fMRI there was an association between ICC_w_ scores and cognitive systems. First, we mapped edgewise scores and then averaged over edges within each of the 17 cognitive systems from the Schaefer 200 layout combined with 21 subcortical regions from Harvard–Oxford atlas. As a trend, connections within a cognitive system for dMRI and resting-state fMRI exhibited the strongest reliability as can be seen from the figure because of the high values along the diagonal (Fig. 1F,G, respectively). However, a direct comparison between dMRI and fMRI showed distinct distribution of reliability across cognitive systems. dMRI reliability was stronger within and between the Temporal Parietal Network, Visual, and Default Mode Networks (Fig. 1H, blue boxes). For the EEG data we could not perform the same mapping to cognitive systems, so instead resting-state ICC_w_ scores from the top 5% ICC_w_ distribution are plotted onto the scalp (Fig. 1I).

**Figure 2:**
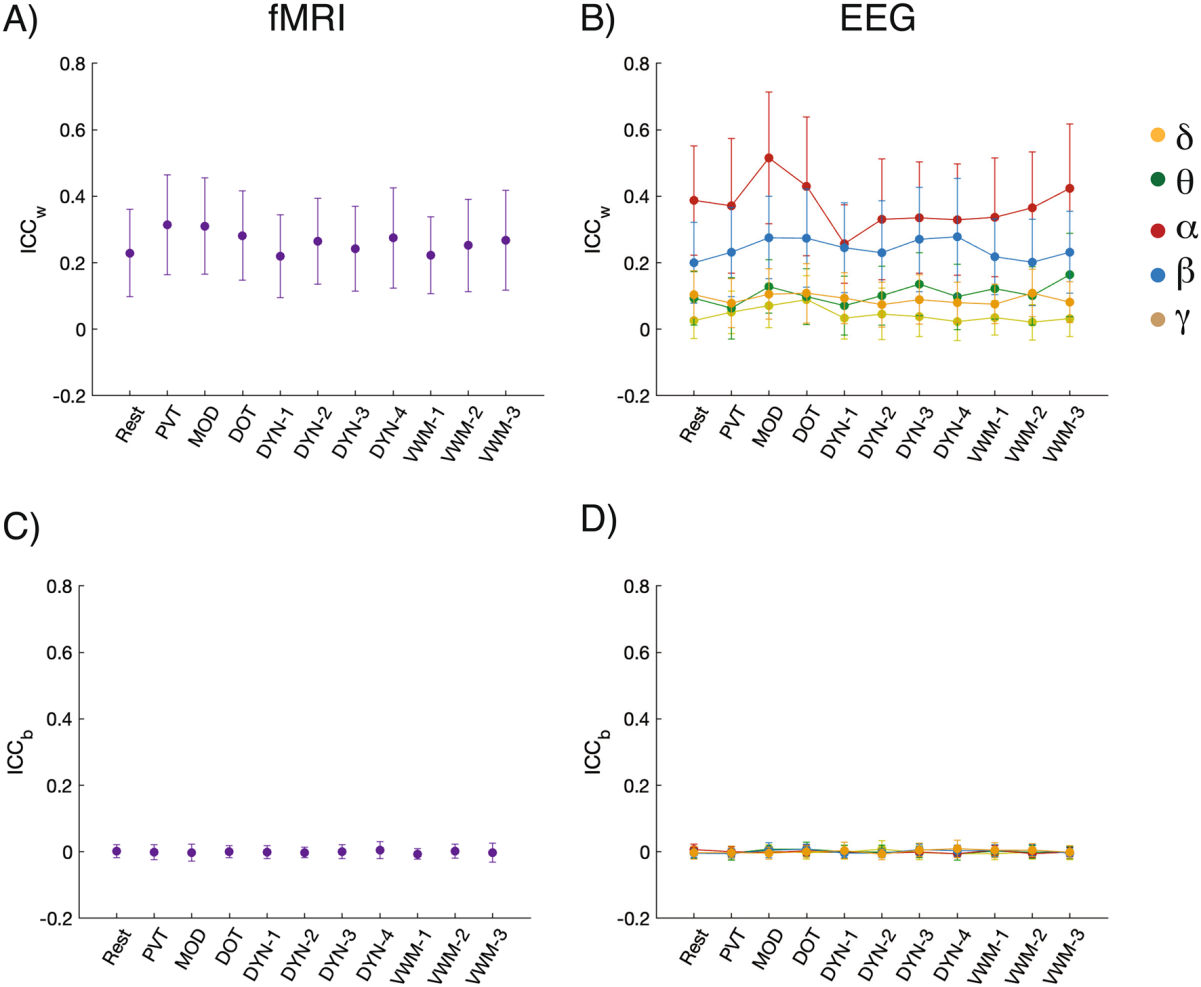
Reliability of individual connections (A and B) within-subjects (ICC_w_) and (C and D) between-subjects (ICC_b_) for fMRI and EEG frequency bands across tasks: DOT, PVT, MOD, VWM-1:3, and DYN-1:4. For each plot, the figure shows the mean and standard deviation. *DOT* dot probe task, *DYN* dynamic attention task, *MOD* modular math task, *PVT* psychomotor vigilance task, *VWM 1–3* visual working memory.

Given the different cognitive demands associated with task performance, one might expect reliability scores during task states to differ from those at rest. However, when we examined task induced changes in reliability, we found that task associated ICC_w_ and ICC_b_ values for fMRI and EEG scores exhibited a similar pattern to resting-state (Fig. 2). We found significant differences when comparing within- and between-subject reliability for fMRI (F_1,501389_ = 1701, p << 0.001, η^2^ = 0.003; one-way ANOVA; Fig. 2A,C) and EEG (F_4,628_ = 2547, p <  < 0.001, η^2^ = 16.2; one-way ANOVA; Fig. 2B,D).

We then focused on the effect a task had on within-subject reliability. For this, we limited our analysis to the ICC_w_ because ICC_b_ values were close to zero. We compared task differences in fMRI using a one-way ANOVA where each task is treated as a variable, and we found significant differences between tasks (F_10,501389_ = 256, p_corrected_ << 0.001, η^2^ = 0.005). It is worth noting that these differences were small, but, for example, resting-state fMRI had consistently lower ICC_w_ values than the other tasks (Fig. 2A). For the EEG, we additionally added each frequency band (δ, θ,α, β, γ) as a variable to the ANOVA design and found that the Task _(Rest, Dot, Mod, Pvt, Dyn1-4, VWM1-3)_ x Frequency_(__δ__,__θ__,__α__,__β__,__γ__)_ interaction was significant (F_40,201190_ = 291, p_corrected_ << 0.001, η^2^ = 0.06). Overall, EEG frequency exhibited larger differences in reliability than task effects, with the α-band having the highest ICC_w_ scores.

**Figure 3:**
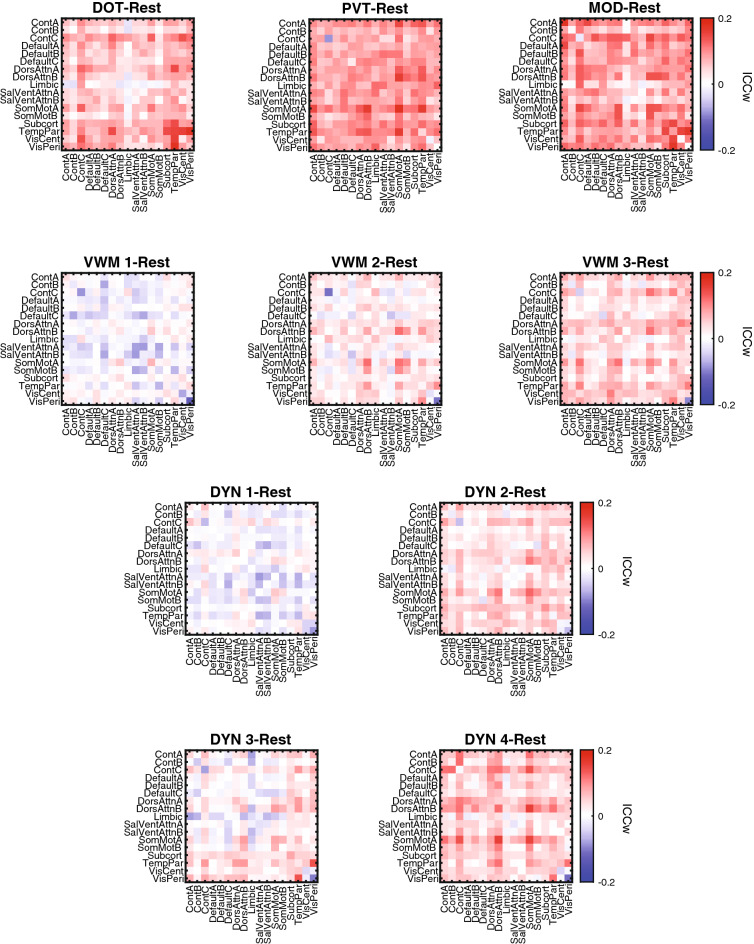
fMRI changes in reliability from resting-state for each task: DOT, PVT, MOD, VWM-1:3, and DYN-1:4. Connections are mapped unto 17 cognitive systems from Schaefer cortical and Harvard–Oxford subcortical atlas. *DOT* dot probe task, *DYN* dynamic attention task, *MOD* modular math task, *PVT* psychomotor vigilance task, *VWM 1–3* visual working memory.

**Figure 4:**
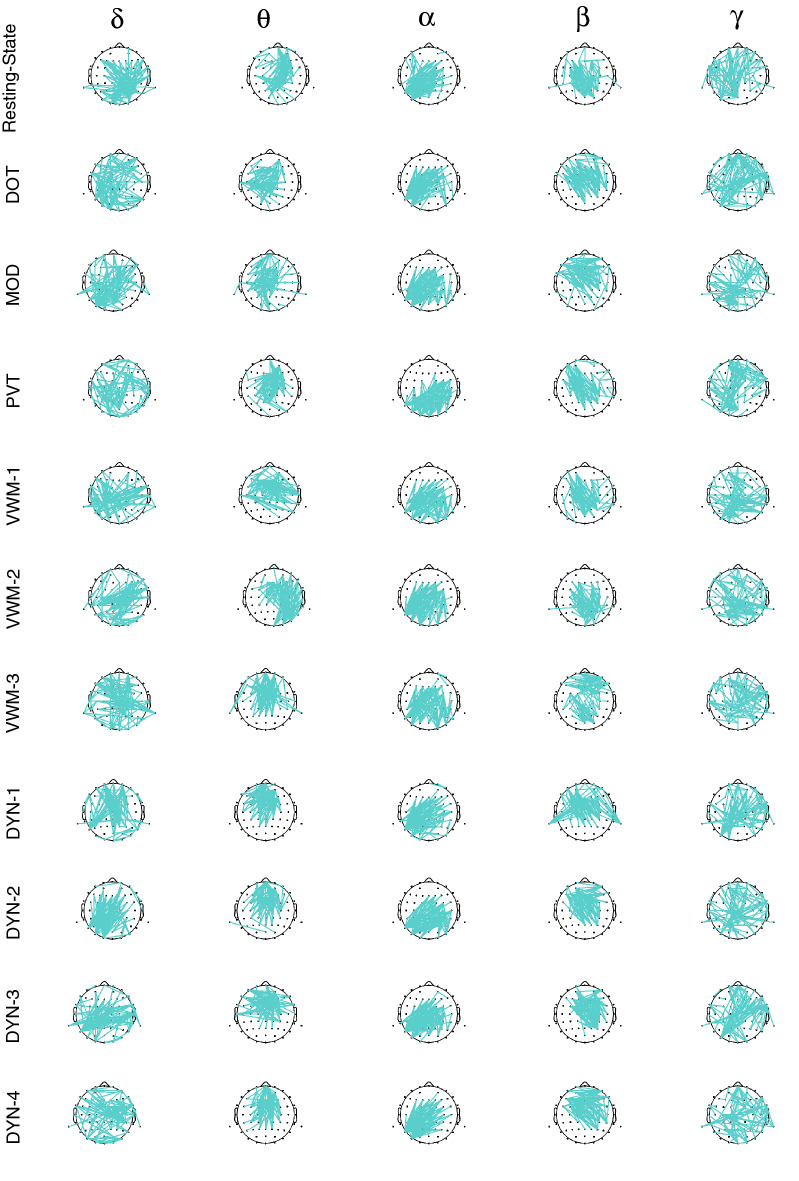
Scalp distribution across tasks for top 5% of ICC_w_ scores. For Resting-State and each task: DOT, PVT, MOD, VWM-1:3, and DYN-1:4, (11 in total), connections with ICC_w_ scores in top 5% for δ, θ, α, β and γ frequency bands are plotted on the scalp. *DOT* dot probe task, *DYN* dynamic attention task, *MOD* modular math task, *PVT* psychomotor vigilance task, *VWM 1–3* visual working memory.

Further, we assessed if for task fMRI there was an association between ICC_w_ scores and cognitive systems. We mapped edgewise scores to the 17 cognitive systems in the same manner as for the resting-state and plotted the difference between the ICC_w_ values during task and resting-state in Fig. 3. Confirming the edgewise results, we generally observed higher reliability during task states. For task EEG data, ICC_w_ scores from the top 5% of the ICC_w_ distribution were plotted onto the scalp and we did not notice any overt reconfiguration in scalp distribution from resting-state to task (Fig. 4).

However, these effects in the fMRI and EEG could be impacted by intra-session reliability^19,20,22^ and motion artifacts in fMRI specifically^6,48^. We estimated the intra-session reliability by splitting the fMRI and EEG data into two halves and separately calculating the functional connectivity for each half of the session. The intra-session similarity between functional connectivity matrices was assessed using the Pearson Correlation. We found moderate intra-session similarity for fMRI data (r > 0.68 for all tasks; Supplementary Fig. S1A) and strong intra-session similarity for EEG data (r > 0.96 for all tasks; Supplementary Fig. S1B).

The lower intra- and inter-session reliability observed in the fMRI data could be due to the limited time of the recordings. Thus, the inter-session fMRI reliability could potentially be increased with more data per subject, as shown in previous work by Gratton et al.^22^ and Laumann et al.^20^, or this issue could be mitigated by combining data across sessions using a “background connectivity” approach^49–51^. However, given that both modalities were recorded for the same amount of time, this suggests that the sampling rate could also have an impact on intra-session reliability. Further, we note that many of the EEG frequency bands have a high intra-session reliability (Fig. S1), yet still show lower ICCw scores than that of the fMRI data (Fig. 1A). This highlights the fact that relationship between inter-and intra-session reliability is complex and potentially modality dependent. Future work should take care when assessing inter-session reliability, especially in data sets with limited time of recordings.

Additionally, since motion can have a significant impact on fMRI functional connectivity, we evaluated how well our preprocessing pipeline accounted for motion. We in general, found weak correlations between motion (as measured using frame displacement) and functional connectivity (Supplementary Fig. S2). Further, since the relationship between motion and functional connectivity has been found to be distance dependent^48^, we evaluated this relation and found no such relation (r < 0.04; all panels in Supplementary Fig. S3).

### Reliability of network measures

We next assessed the reliability of higher order network properties. For each brain network, nine measures were calculated along with their corresponding ICC_w_ and ICC_b_ scores. Since the functional connectivity was estimated on 200 regions for the fMRI and 62 sensors in the EEG, this difference could be a confound when comparing between the two modalities. To more closely match the number of EEG sensors, we estimated fMRI functional connectivity and network measures using the Desikan-Killiany brain atlas which contains 68 regions (fMRI68).

**Figure 5:**
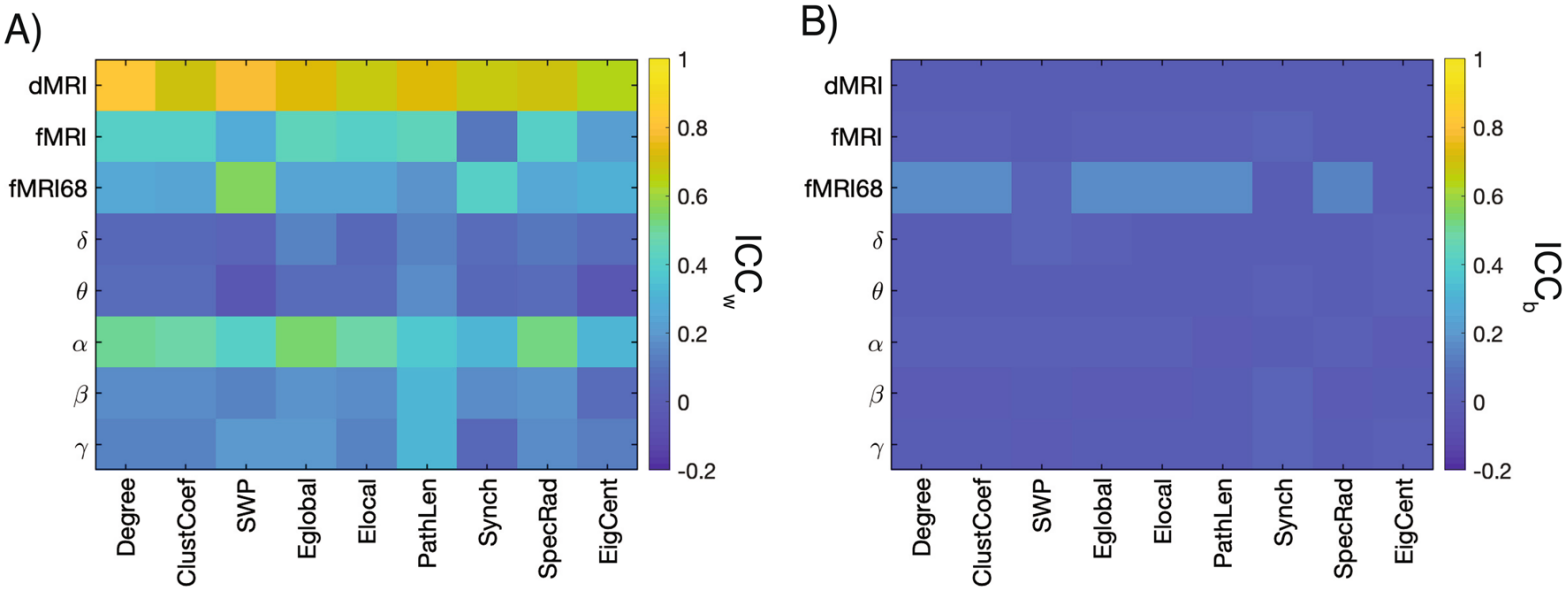
Graph Measures for dMRI and Resting-State fMRI and EEG for (**A**) ICC_w_ and (**B**) ICC_b_ values. For fMRI data, the analysis was conducted using both the Schaefer 200 (fMRI) and Desikan-Killiany (fMRI68) atlases.

We first assessed differences among ICC_w_ and ICC_b_ scores using a one-way ANOVA where all network measures across imaging modalities were pooled together and grouped based on whether they are a within- or between-subject measure. As expected, we found stronger values for the ICC_w_ compared to the ICC_b_ (F_1,125_ = 104, p_corrected_ << 0.001, η^2^ = 0.83; Fig. 5).

**Figure 6:**
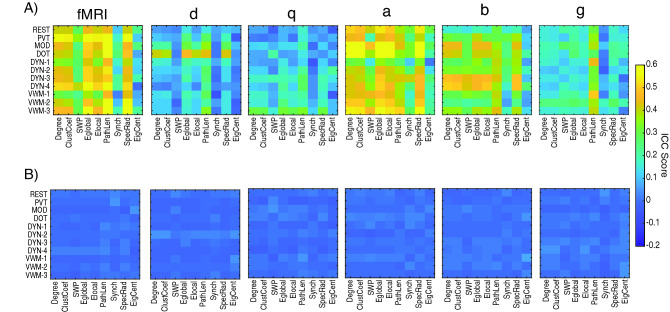
ICC values for network measures across task and resting-state. (**A**) ICC_w_ and (**B**) ICC_b_ values across tasks for fMRI and EEG frequency bands.

Focusing on the within-subject measures, as shown in Fig. 5A, across all imaging modalities and network properties, the dMRI exhibited the highest ICC_w_ scores [0.71 ± 0.06 (SD)]. By comparison, resting-state fMRI exhibited relatively poor reproducibility [fMRI: 0.35 ± 0.12 (SD); fMRI68: 0.30 ± 0.11 (SD)], and the EEG reproducibility was frequency dependent with the α-band having the highest ICC_w_ scores [0.43 ± 0.09 (SD)]. These results were tested with a one-way ANOVA in which dMRI, fMRI, fMRI68, and each EEG frequency bands (δ, θ,α, β, γ) were treated as separate groups, and we found significant differences between them (F_7,62_ = 65, p_corrected_ << 0.001, η^2^ = 7.33; Fig. 6A). The same analysis for the ICC_b_ scores found significant difference across all modalities (F_7,62_ = 15, p_corrected_ <  < 0.001, η^2^ = 1.69; Fig. 5A), but these differences were driven by the fMRI68 having a stronger ICC_b_ [0.11 ± 0.08 (SD)] whereas the other modalities had ICC_b_ close to zero (Fig. 5B). These ICC_b_ results suggest that, for fMRI, the choice of atlas could be an important factor in identifying consistent measures across subjects.

We then asked if performing a task alters the reliability of network measures (Fig. 6). To evaluate how a task alters the within- and between-subject reliability, for fMRI we designed an ANOVA that assessed changes across tasks, network measures, and ICC. We found significant effects for Task x ICC (F_8,80_ = 99, p_corrected_ << 0.001, η^2^ = 9.90) and Network Measure x ICC (F_10,80_ = 9.88, p_corrected_ << 0.001, η^2^ = 0.12; Fig. 6A). For the EEG, we added frequency as a factor to the ANOVA design and evaluated a Task x Frequency x Network Measure x ICC ANOVA design, and we found a significant interaction between Task x Frequency (F_32,792_ = 5.35, p_corrected_ < 0.001, η^2^ = 0.22) and Frequency x Network Measure (F_40,792_ = 6.33, p_corrected_ < 0.001, η^2^ = 0.32; Fig. 6A). From Fig. 6 it is apparent that the α-band is the most consistent across resting- and task-state, while the β-band shows an increase in ICC_w_ in the task-states. It is also worth noting that Synchronizability and Eigenvector Centrality exhibited weaker ICC_w_ scores relative to the other metrics across resting- and task-states for both fMRI and EEG.

Further, we conducted the intra-session half-split similarity analysis for the network measures. Overall, we found stronger half-split similarity values across the EEG frequencies for each network measure and task when compared to half-split similarity values for the fMRI (Fig. S4). It is worth noting that the half-split reliability was lower for small-world propensity and synchronizability, reflecting their lower ICC values.

### Fingerprinting analysis

**Figure 7:**
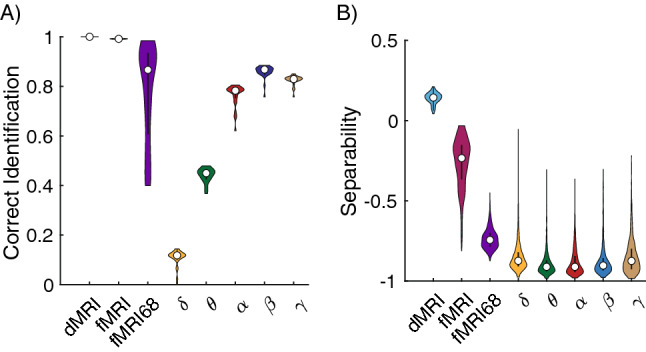
Fingerprinting performance across imaging modalities. (**A**) Proportion of networks that were correctly matched to the corresponding individual for dMRI, fMRI, and EEG derived brain networks. (**B**) Separability of each network in being matched to corresponding individual. fMRI indicates results using the Schafer 200 atlas whereas fMRI68 indicates results using the 68 regions Desikan-Killiany atlas. For each violin plot, the central dot indicates the median, and the line indicates the 25th–75th percentiles.

Our analysis so far has confirmed that dMRI networks are more reliable within a subject than fMRI and EEG networks. Therefore, we expect that dMRI networks will have a higher probability of being able to identify an individual from a group, similar to a fingerprint^39^. For functional networks, we would similarly expect the same for the α-band EEG, given the relatively higher reliability scores. In order to fingerprint an individual, brain networks from the individual should be more similar to each other across runs relative to networks obtained from other individuals. To formally assess the similarity between brain networks, we measured the similarity using the Euclidian distance (“Materials and methods”). Our results indicate that fingerprinting was not uniform across all derived networks (F_7,236_ = 285, p_corrected_ << 0.001, η^2^ = 8.45). As expected, structural dMRI networks had the highest accuracy. However, the performance of fMRI networks was dependent on the number of regions, with connectivity based on the Schaefer 200 brain regions outperforming the 68 regions from the Desikan-Killiany atlas. Additionally, both fMRI atlases performed better than α-band EEG derived networks, despite the α-band exhibiting stronger reliability values. In fact, in the EEG, the β-band networks had the highest fingerprinting accuracy (Fig. 7A).

However, this analysis does not tell us about the separability across the networks derived from the different imaging modalities. Here, we define separability as the difference in similarity between the minimum within-subject value for a network to the maximum between subject similarity for that network (see Materials and methods). Therefore, positive separability values indicate that a particular network for an individual is always more similar to other networks from that individual and negative values indicate the opposite. Separability values across imaging modalities were found to be significantly different (F_7,10093_ = 7898, p_corrected_ << 0.001, η^2^ = 5.48). In addition, despite dMRI and fMRI having similar accuracy in fingerprinting, dMRI networks were more separable than fMRI and EEG [dMRI: 0.14 ± 0.04 (SD); fMRI: -0.26 ± 0.27 (SD); fMRI68: − 0.74 ± 0.06 (SD); δ, θ, α, β and γ: < -0.85 (mean)] (Fig. 7B).

## Discussion

In the current work, we analyzed the reproducibility of multimodal and multi-task structural and functional brain networks in a unique longitudinal and multi-modal dataset with simultaneous EEG-fMRI recordings. In our analysis, each subject contained brain networks derived from dMRI, fMRI and EEG data, allowing us to assess how reliability differed in brain networks derived from different modalities and across task states.

We first assessed the reliability of individual connections in the structural and functional brain networks and found stronger within- than between-subject reliability across all imaging modalities, in line with previous results^12,13,24,52–54^. The most reliable connections were also the ones that tended to be the strongest, corroborating previous findings in fMRI networks^12,24^. In addition, when mapped onto cognitive systems, these connections exhibited distinct patterning. As a trend, for dMRI and resting-state fMRI, connections within a cognitive system exhibited the strongest reliability, consistent with previous studies in functional networks^12,52–54^. However, a direct comparison between dMRI and resting-state fMRI showed distinct distribution of reliability across cognitive systems. dMRI reliability was strongest within the Frontal-Parietal Control system and between the Visual to Default Mode and Temporal Parietal system, while in resting-state fMRI stronger values were distributed between cognitive systems.

When assessing task mediated changes, we found an increase in reliability across most tasks relative to resting-state in fMRI networks. In addition, we observed an increase in this reliability across multiple sessions of a given task, potentially indicative of an effect of learning the task. This finding complements results from a previous study that found adding task-state fMRI networks improves predictive outcomes relative to resting-states fMRI^55^. Here, we take care to note that the patterns of functional activity observed during a given task are expected to be different than those observed during rest, as they are likely the result of co-occurring BOLD responses across different regions of the brain that are specific to performing the task. As such, we also expect the patterns to be different between tasks that invoke different brain circuitry. While some studies consider these task-dependent signals in functional connectivity to artificially increase the observed underlying relationships between regions^56^, here, we instead want to emphasize the observed differences between task-dependent patterns of functional connectivity, noting that the specific task being performed can differentially affect reliability measurements of task-dependent functional connectivity.

For EEG, the α- and β-bands had the highest reliability scores for both resting- and task-states, confirming previous results^26^. The strong reliability for the α- and β-band could be due to the fact that these frequencies are consistently active, while the other frequency bands tend to have transient activity. In a similar manner to fMRI, EEG reliability increased during a task, but this increase was primarily in the α- and β-bands. In addition, we found no major changes when we mapped connections on the scalp from resting-state to task-state. This could be due to the low spatial resolution of EEG^57^. Additionally, an inherent limitation of such EEG-fMRI data is the higher-than-normal noise in the EEG, thus requiring a larger number of channels to be interpolated. As a result, this could affect the reliability results in the EEG signal.

When examining the reliability of higher order network properties, we found that network properties had overall stronger reliability scores than individual connections in line with previous findings of Braun et al.^21^. This might lead one to ask how the prevalence of low reliability scores across most connections could produce fair to excellent reliability in higher order network properties? This result could be due to the fact that edges with higher reliability scores are associated with the stronger connections. Our graph theoretical properties are dependent on connection strength, and the stronger the connection, the more variance it accounts for in the higher order network values. Thus, despite most connections having poor reliability, the few strong connections with good to excellent reproducibility have a disproportionately higher impact on the reliability of a network measure. The notable exception is that in fMRI and EEG, synchronizability and eigenvector centrality had lower reliability scores than the other network properties. One possible reason for this is that these measures, particularly eigenvector centrality, are very sensitive to the state of the subject^58^. These results indicate these measures might be more sensitive to detecting meaningful differences between individuals in studies where one is attempting to link functional brain connectivity to task performance or behavior.

We also found task associated differences in reliability for the fMRI and EEG. However, for the EEG, the strongest increases in reliability were in the α- and β-bands. However, in contrast to Deuker et al.^18^ we did not find a corresponding increase in ICC_w_ scores in the δ and θ bands with task.

We found that dMRI and fMRI outperformed EEG derived networks in fingerprinting. However, the separability was not equal across these networks, with dMRI outperforming all functional networks. This is likely due to the fact that, unlike functional connectivity, structural connectivity is not state dependent. Also, despite the fact that the α-band EEG showed higher reliability, all EEG frequency bands performed worse than fMRI in our fingerprinting analysis. This is likely driven by the fact that the fMRI data has a larger number of strong connections than EEG data, and these strong connections will have a greater impact in the similarity calculations used in the analysis.

It has been found that brain activity measured with fMRI is stable over time^20,22,59,60^ and in fMRI, within-subject variance can be reduced with high quality data with long scan times (~ 15 min) and multiple sessions^20,24,53,61^. It has been argued that large amounts of data are needed in order to differentiate between true and artifact induced variance^6,19^ and previous studies have found that reliability increases with more data^12,20,53,62,63^. This high quality data is important because Horien et al.^60^ found that motion characteristics can be unique to an individual and can fingerprint a subject at a level greater than chance. In our data, individual scan times were limited to approximately 5 min, but data was collected over multiple sessions for a relatively large number of subjects, suggesting that we might expect more reliable results. However, our observation of the relatively weak accuracy and separability of EEG (a more direct measure of neuronal activity than fMRI) in fingerprinting an individual raises questions as to whether the increase in fingerprinting performance in fMRI on long time scans is based on neuronal activity. Also, respiration induced artifacts in fMRI exhibit the same stability over time^64^, which could also lead to increased reliability measurements.

Our direct comparison of fingerprinting between structural and functional networks indicates that structural networks are more sensitive. In addition, these results indicate that the patterning in structural connectivity is far more unique to an individual than those in corresponding functional networks. These results suggest that structural networks might have more discriminative power than functional networks.

Unique brain connectivity features have previously been proposed to play a role in differences underlying behavior and cognition^65^. Specifically, difference in behavioral performance in motor and decision associated tasks are correlated with fractional anisotropy of the corpus callosum^66,67^, optic radiation^68^ and grey matter density^69^. Cortical thickness within the superior parietal lobes has been found to be correlated with the rate of switching in a perception based task^70^. In addition, structural features unique to an individual lead to characteristic brain functional activity in modeling analysis and task performance^38,71^.

Is a connection with poor reliability good or bad? To answer this, we need to be mindful of the goal at hand. First and foremost, we need to make sure that reliability values are not due to noise in the signal or artificially low due to short lengths of recordings. On the other hand, if we are confident that low reliability is a genuine part of the signal, then that is also a very informative finding. The seminal work of Poldrack et al.^72^ found that functional connectivity exhibits a high level of variability within the same person over the course of a year. Along these lines, Noble et al.^61^ found that functional connections with strong reliability are not very informative when it pertains to predicting behavior. However, we need to be mindful that this is an effect limited to functional connectivity. Therefore, structural connections and/or higher-order network metrics might exhibit a stronger association between reliability and behavior. Also, finding highly reproducible brain connections and/or measures might be very important if we are looking for deviations from expected values that could be used as biomarkers for disease identification/progression. Alternatively, connections and/or measures with low reliability might be useful for studying individual differences and making correlations between structure and performance/behavior.

But, even beyond reliability and noise, our functional results could, along with previous literature, reflect the natural day-to-day changes in our brain. Neuroplastic changes in the brain are the hallmark of learning and memory^73^, and these changes or natural fluctuations and modifications in the *neural code*^74^, reflecting learning and memory could be reflected in functional connectivity. Indeed, there are many examples of rapid neuroplastic changes in the brain that results in functional connectivity changes (e.g., Nierhaus et al., 2019), but see Perich et al.^76^ as an alternative theory. Moreover, in this particular dataset, individuals were recruited to capture substantial variability in sleep without experimental manipulation. While there is a substantial literature on brain related decrements due to sleep deprivation^77,78^ little is known about naturalistic fluctuations in sleep^46,79^. These individuals instead could be more “plastic” (or “stationary”) than other individuals. Future studies will hopefully work to disentangle the effects of neuroplasticity from experimental factors that affect reliability estimates. 

fMRI-based analysis has been around for over two decades, but its clinical use has been limited, raising questions about its usefulness as a diagnostic tool. In addition, given that the effectiveness of any diagnostic tool is only as useful as it can be applied to an individual, then in this regard, structural networks should take a more prominent role in medicine. Regardless, one must consider how measures of reliability relate to the modality being studied, the state of the brain, and the question at hand in order to meaningfully ask questions about how brain networks change with disease or how individual differences in structure relate to performance and behavior.

## Materials and methods

### Participants

The University of California, Santa Barbara (UCSB) Human Subjects Committee (#16-0154) and Army Research Laboratory Human Research Protections Office (#14-098) approved all procedures, and all participants provided informed written consent. Research was conducted in accordance with the declarations of Helsinki. The data presented in this manuscript represent a subset of data collected as part of a large-scale, longitudinal experimental that collected bi-weekly structural and functional brain data. A full description of the study can be found in^46^. Here we analyze data from 27 healthy participants who were recruited by word of mouth and local advertisements. Note that by study design, participants were excluded from the multi-session segment of the study if they did not experience sleep variability. Data is accessible upon request as far as allowed by the security policy and guidelines established with the ethics committee of the US Army Research Laboratory Human Research Protection Program.

### Data description

Over the course of 16 weeks, subjects were asked to complete 8 recording sessions involving dMRI and simultaneous EEG-fMRI. For each session, simultaneous EEG-fMRI recording consisted of a 5-min resting state and 10 tasks with varying levels of cognitive demand; specifically:

- Dot Probe Task (Dot)^80^;
- Dynamic Attention Task (DYN 1–4) with four repetitions of the same task^81^;
- Modular Math (MOD)^82^;
- Psychomotor Vigilance Task (PVT)^83^, and;
- Visual Working Memory (VWM 1–3) with three repetitions of the same task^84^.

Table 1 shows the number of subjects and sessions included in the analysis for each imaging modality and task. Although 27 participants were included in the data set, not all participants participated in all 8 imaging sessions, and some subjects’ fMRI data was excluded due to artifacts. We therefore selected 25 subjects with all 8 sessions of dMRI data for analysis, and for EEG-fMRI data, we analyzed only six sessions of data in order to make a trade-off between maximizing the number of subjects and number of sessions in our data set. As shown in Table 2, for the fingerprinting analysis using dMRI data, we again used 25 subjects, all of which had an equal number of sessions (8 sessions). For the fingerprinting analysis using fMRI data, 15 subjects were included with all 6 sessions of resting-state and task recordings, and for the EEG data, we used 26 subjects with resting-state and all tasks over 6 sessions.

**Table 1:** Number of subjects, sessions, task length, and inter-session interval.

Task	dMRI subject	EEG subject	fMRI subject	Session	Time (min)	Interval (days)	Bad channels
n/a	25	n/a	n/a	8	n/a	14	n/a
Resting-state	n/a	27	26	6	5	14	6.7
DOT	n/a	27	25	6	14	14	7.5
DYN-1	n/a	27	27	6	5	14	6.6
DYN-2	n/a	27	27	6	5	14	6.5
DYN-3	n/a	27	26	6	5	14	6.5
DYN-4	n/a	27	20	6	5	14	6.5
MOD	n/a	27	17	6	14	14	8.2
PVT	n/a	27	19	6	12	14	7.5
VWM-1	n/a	27	27	6	6	14	6.6
VWM-2	n/a	27	23	6	6	14	7.3
VWM-3	n/a	27	17	6	6	14	7

**Table 2:** Number of subjects and sessions for fingerprinting analysis.

Modality	Subjects	Sessions	Task included
dMRI	25	8	n/a
fMRI	15	6	Yes
EEG	27	6	Yes

### fMRI acquisition and preprocessing

Functional neuroimaging data were acquired on a 3 T Siemens Prisma MRI using an echo-planar imaging (EPI) sequence (3 mm slice thickness, 64 coronal slices, field of view (FoV) = 192 × 192 mm, repetition time (TR) = 910 ms, echo time (TE) = 32 ms, flip angle = 52º, and voxel size: 3 × 3 × 3 mm). For repeated scans, a T1-weighted structural image was also acquired using a high-resolution magnetization prepared rapid acquisition gradient echo (MPRAGE) sequence (TR = 2500 ms, TE = 2.22 ms, and FoV = 241 × 241 mm with a spatial resolution of 0.9 × 0.9 × 0.9 mm), for use in coregistration and normalization.fMRI BOLD images were preprocessed using Advanced Normalization Tools (ANTs)^85^. Physiological artifacts including respiration and cardiac cycle effects were corrected using the retrospective correction of physiological motion effects method, RETROICOR^86^, implemented in MEAP v1.5^87^. Head motion was estimated using antsMotionCorr, and the motion correction was completed as follows: (1) An unbiased BOLD template was created within each session by averaging the motion-corrected BOLD time series from each run. (2) The BOLD templates were coregistered to the corresponding T1-weighted high resolution structural images, collected in each session. (3) Each session was spatially normalized to a custom study-specific multi-modal template which included T1-weighted, T2-weighted and GFA images from twenty-four quasi-randomly selected participants chosen to match the study population. (4) The template was then affine-transformed to the coordinate space of the MNI152 Asymmetric template. (5) Finally, the fMRI volumes were transformed using the estimated head motion correction, BOLD template coregistration, BOLD-to-T1w coregistration and spatial normalization into MNI space using a single Hamming weighted sinc interpolation. After these transformations, the final step in the preprocessing was to extract time-series from fMRI scans for functional connectivity analyses. Two atlases were used to reduce the 3D volume data into 221 nodal time series data: (1) the cortical Schaefer 200 atlas^88^ which was derived from intrinsic functional connectivity in resting state fMRI and (2) 21 subcortical regions from the Harvard–Oxford atlas based on anatomical boundaries^89^. As the atlases are in MNI coordinate space, voxels within each labelled region of the atlases were simply averaged, and time series were extracted for the following connectivity analyses.

To assess functional connectivity among ROIs, mean regional time-courses were extracted and standardized using the nilearn package^90^ in Python 2.7, and confound regression was then conducted. In particular, the time series for each region was detrended by regressing the time series on the mean as well as both linear and quadratic trends. There were a total of 16 confound regressors, which included: head motion, global signal, white matter, cerebrospinal fluid and derivatives, quadratics and squared derivatives. This functional connectivity preprocessing pipeline was selected based on conclusions from prior work that examined performance across multiple commonly used preprocessing pipelines for mitigating motion artifact in functional BOLD connectivity analyses^48,91^.

To construct the fMRI networks, the signal from all voxels within a brain region were averaged, and the Pearson Product Correlation (*R*) between two brain regions was calculated as

1 $$R= \frac{cov(x,y)}{\sqrt{{\sigma }_{x}{\sigma }_{y}}},$$

where *x* and *y* represent the time-series data from two different regions and σ is the variance of the time series. To account for negative correlations, the absolute value of the correlations was used to construct weighted functional connectivity matrices.

### EEG acquisition and preprocessing

Continuous EEG recordings were captured simultaneously with an fMRI-compatible EEG equipped with standard Ag/AgCI electrodes from 64 sites on the scalp oriented in a 10–20 scheme system (Brain Products, Gilching, Germany). Initial fMRI pulse and ballistocardiographic artifact correction was completed in BrainAnalyzer 2 (Brain Products, Gilching, Germany) using classic subtraction and filtering approaches^92,93^. These mid-level processed EEG measurements were then further processed using in-house software in MATLAB (Mathworks, Inc., Natick, MA, USA) and the EEGLAB toolbox^94,95^. Despite the subtraction and filtering approaches applied, residual artifact from the fMRI pulse persisted. To remove these lingering artifacts, we developed a new cleaning pipeline.

Our cleaning pipeline included steps tailored to remove common EEG artifact (e.g., eye blinks, muscle-related activity) and then targeted the high frequency noise in the 16–19 Hz and 34–38 Hz range. EEG data were bandpass filtered between 0.75 and 50 Hz using a Finite Impulse Response (FIR) filter. Next, EEGLAB’s automated clean_rawdata function was used to determine channels that differed substantially from the estimated signal (derived from other channels) or had consistent flat-lining. Then, the EEG data were subjected to an Independent Component Analysis (ICA) decomposition and the ADJUST algorithm^96^ was used to remove ICA components associated with stereotyped noise. Following ICA decomposition, bad channels were interpolated using spherical interpolation. As a final step in EEG preprocessing, the EEG data were subjected to Artifact Subspace Reconstruction (ASR)^97,98^, which we used to target the aforementioned residual high frequency noise from the fMRI artifact. This method, in combination with the ICA cleaning method allows for the targeting of both stationary and non-stationary persistent artifacts. To deploy ASR on the dataset, we first created a “clean” reference signal from each subject’s EEG data by: 1) concatenating EEG segments that were at least 1000 ms long with amplitude below 100 µV, (2) and notch filtering (FIR) the EEG between 16–19 and 34–38 Hz. Following the creation of the reference signal, ASR was then used to reconstruct the EEG that contained large fluctuations greater than 5 standard deviations beyond the reference signal (in 500 ms chunks). Lastly, the data were re-referenced to a common average reference.

To construct EEG networks, the signal from each sensor was separated into standard frequency bands corresponding to δ (1–3 Hz), θ (4–7 Hz), α (8–13 Hz), β (15–30 Hz) and γ (30–60 Hz) with a Butterworth filter (8th order) followed by Hilbert transformation. Weighted functional connectivity adjacency matrices were constructed for each frequency band using the de-biased weighted phase-lag index (dwPLI)^47^. Each node in the adjacency matrix corresponds to a channel with the weight representing the strength (phase-lag) of the connection. Specifically, dwPLI is calculated as,

2 $$dwPLI= \frac{{\sum }_{i=1}^{N}{\sum }_{j\ne i}I\left({\mathrm{X}}_{\mathrm{i}}\right)I({\mathrm{X}}_{j})}{{\sum }_{i=1}^{N}{\sum }_{j\ne i}|I\left({\mathrm{X}}_{\mathrm{i}}\right)I\left({\mathrm{X}}_{j}\right)|},$$

where *I(X*_*i*_*)* corresponds to the imaginary component of time series data (*X*) from channel *i*. Thus, dwPLI is the sum of all pairwise products of the magnitudes of the imaginary components and accounts for any bias due to the number of data points.

### dMRI acquisition and preprocessing

Diffusion spectrum imaging (DSI) scans were acquired for each session. DSI scans sampled 258 directions using a Q5 half-shell acquisition scheme with a maximum b-value of 5000 and an isotropic voxel size of 2.4 mm. Minimal preprocessing was carried out on the DSI scans and was restricted to motion correction. Following a similar procedure to the fMRI motion correction, motion was first assessed and applied for all of the b0 volumes, and a template was created for each scan composed of the average of the b0 volumes. Next, the b0 volumes and vectors were transformed using the estimated head motion correction, b0 template coregistration, b0 template-to-T1w coregistration and spatial normalization into MNI space using a single Hamming weighted sinc interpolation.

Fiber tracking was performed in DSI Studio (www.dsi-studio.labsolver.org) with an angular cutoff of 35°, step size of 1.0 mm, minimum length of 10 mm, spin density function smoothing of 0, and a maximum length of 250 mm. Deterministic fiber tracking was performed until 500,000 streamlines were reconstructed for each session. As with the fMRI volume data, streamline counts were estimated in 200 nodes using the same Schaefer 200 atlas^88^ and 21 subcortical regions part of the Harvard–Oxford atlas^89^. Connectivity matrices were then normalized by dividing the number of streamlines (*T*) between region *i* and *j*, by the combined volumes (*v*) of region* i* and *j*,

3 $${W}_{ij}=\frac{{T}_{ij}}{{v}_{i}+{v}_{j}}.$$

### Graph theoretical analysis

We calculated nine commonly used and diverse graph metrics on each weighted dMRI, fMRI and EEG network. The graph metrics are: degree, clustering coefficient, characteristic path length, small-world propensity, global and local efficiency, synchronizability, spectral radius, and eigenvector centrality. See supplemental for detailed description of each network measure.

### Degree

The weighted node degree (*k*_*i*_) is defined as the sum of all connections of a node^99^,

4 $${k}_{i}= {\sum }_{j \in N}{W}_{ij},$$

where *W* is the weighted adjacency matrix of a network with *N* nodes.

### Clustering coefficient

The weighted clustering coefficient (*C*) for node *i* is the intensity of triangles in a network^100^ and is calculated as,

5 $${C}_{i}= \frac{1}{{b}_{i}({b}_{i}-1)}\sum_{j,h}{({W}_{ij} {W}_{ih} {W}_{jh})}^{1/3},$$

where *W* is the weighted adjacency matrix and *b* is the number of edges for node *i*.

### Characteristic path length

The characteristic path length (*L*) is the average shortest path length between all nodes^99^,

6 $$L= \frac{1}{N} {\sum }_{i\in N}\frac{{\sum }_{j\in N,j\ne i}{d}_{ij}^{w}}{N-1},$$

where ${d}_{ij}^{w}$ is the is the distance between nodes i and j. To calculate ${d}_{ij}^{w}$, we first take the inverse of the edge weights to transform the weight to a measure of length (i.e., to transform a strong connection strength to a short length). We then determine the shortest path between nodes *i* and *j* (using the inverted weights), and ${d}_{ij}^{w}$ is the sum of the inverse of the edge weights along this shortest path.

### Small-world propensity

Small-world propensity (φ) quantifies the extent to which a network displays small-worldness, a network property that combines the presence of local clustering with a short path length, while factoring in variation in network density^101^. Small-worldness is calculated as,

7 $$\phi =1- \sqrt{\frac{{\Delta }_{C}^{2}+{\Delta }_{L}^{2}}{2},}$$

8 $${\Delta }_{C}= \frac{{C}_{latt}-{C}_{obs}}{{C}_{latt}-{C}_{rand}},$$

9 $${\Delta }_{L}= \frac{{L}_{obs}-{L}_{rand}}{{L}_{latt}-{L}_{rand}},$$

where *C*_*obs*_ is the observed clustering coefficient and *L*_*obs*_ is the observed characteristic path length of the network; *C*_*latt*_, *L*_*latt*_*, C*_*rand*_*,* and L_rand_ are clustering coefficient and characteristic path length from lattice and random networks with the same number of nodes and edge distribution.

### Global and local efficiency

The efficiency of a node is the inverse of the path length^99^. Global efficiency (*E*_*g*_) is the inverse shortest path length,

10 $${E}_{g}= \frac{1}{N} {\sum }_{i\in N}\frac{{\sum }_{j\in N,j\ne i}({d}_{ij}^{w}{)}^{-1}}{N-1},$$

where ${d}_{ij}^{w}$ is the previously defined distance between node *i* and *j*.

Local efficiency (*E*_*l*_) is the global efficiency computed on the neighborhood of node *i*,

11 $${E}_{l}= \frac{1}{N} {\sum }_{i\in N}\frac{{\sum }_{j,h\in N,j\ne i} ({w}_{ij}{w}_{ih}[{d}_{jh}^{w}{({N}_{i})]}^{-1}{)}^{1/3}}{{k}_{i}({k}_{i}-1)},$$

where* w*_*ij*_ and* w*_*ih*_ is strength of the connection between node *i* to *j* and *h*, respectively, and *d*_*jh*_* (Ni)* is the length of the shortest path between nodes *j* and* h* that contains only neighbors of node *i*.

### Synchronizability

Synchronizability is a measure of linear stability for a network of coupled dynamical systems^102^,

12 $$S= \frac{{\lambda }_{2}}{{\lambda }_{n}},$$

where *λ*_*2*_ is the second smallest eigenvalue of the unnormalized Laplacian matrix (*L*) and* λ*_*n*_ is its largest eigenvalue. The Laplacian is calculated as,

13 $$L=D-W,$$

where *D* is the degree matrix of the weighted adjacency matrix, *W*.

### Spectral radius

The spectral radius measures the ease with which diffusion process can occur in a network. The spectral radius is calculated as,

14 $$\rho \left(W\right)=\mathrm{max}\left\{\left|{\lambda }_{1}\right|,\dots ,\left|{\lambda }_{n}\right|\right\},$$

where |*λ*| corresponds to the absolute value of the eigenvalues of a network.

### Eigenvector centrality

Eigenvector centrality (*EC*_*i*_) measures how influential a node is in a network, with a high value indicating a node is connected to other highly influential nodes^103^. The eigenvector centrality of node i is given by the i-th entry in the dominant eigenvector, which is the vector ***v*** = [*v*_*1*_*,…v*_*N*_] that solves

15 $${\lambda }_{1}v= W{v}^{T},$$

where ${\lambda }_{1}$ is the largest eigenvalue of the weighted adjacency matrix, *W*.

### Intra-class correlation

The intra-class correlation (ICC) is a measure used to quantify the test–retest reliability of a measure. We used the ICC to measure the consistency of individual connections across the dMRI, fMRI and EEG networks and across the graph metrics for each network. To accomplish this, we calculated two variants of the ICC, the within (ICC_w_)- and between (ICC_b_)-subjects^104^. ICC_w_ and ICC_b_ are, respectively, calculated as,

16 $${ICC}_{w}=\frac{I(RMS-EMS)}{J*SMS+I*RMS+\left(IJ-I-J\right)EMS} ,$$

17 $${ICC}_{b}=\frac{J(SMS-EMS)}{J*SMS+I*RMS \left(IJ-I-J\right)EMS} ,$$

where *I* is the number of subjects and *J* is the number of sessions, *SMS, RMS and EMS* represent the ANOVA measures of mean square error between sessions, subjects, and due to error, respectively. The reliability of a measurement is considered: (1) “poor” if the ICC values is less than 0.4; (2) “fair” for ICC values between 0.4 and 0.6; (3) “good” for ICC values between 0.6 and 0.8; and (4) “excellent if ICC values exceed 0.8.

### Fingerprinting analysis

To perform a fingerprinting analysis, as in Finn et al., 2015, we quantified the degree of similarity between networks. This analysis was performed separately for each of the dMRI, fMRI and EEG modalities. For each individual, connectivity matrices were converted for each individual and run into a vector using the values from the upper triangle of the matrix resulting in vectors of 1 × 24,310 for dMRI and fMRI, and 1 × 2016 for EEG. Thus, each vector, *p*, represents a single connectivity matrix for a given subject during a given session, and for functional matrices, in a given state (task/rest).

Next, separately within each modality, for each connectivity matrix (representing a subject, session, and state), we calculated the pairwise similarity between two vectors,* p* and *q*, using the Euclidian distance to create a dis-similarity matrix (*D*), where

18 $${D}_{pq}= \sqrt{\sum {( p-q)}^{2}} ,$$

and each entry in *Dpq*, corresponds to the dis-similarity between the brain network *p* to *q*. However, since the Euclidian distance formally assesses dis-similarity and we were interested in evaluating similarity, we converted from a dis-similarity to a similarity (*S*) measure by

19 $${S}_{pq}=\frac{max\left(D\right)- {D}_{pq}}{max\left(D\right)},$$

where *max(D)* corresponds to the largest value in matrix *D*. This normalization ensures that the similarity matrix *S* $\in$ [0 1].

In order to perform a fingerprinting analysis, for each vector, *p*, we then looked for the entry *S*_*pq*_ with the highest similarity value. If for this entry, the vectors *p* and *q* were from the same individual (but could be from different sessions or states), then the fingerprinting analysis was classified to be successful at identifying the individual.

Fingerprinting performance for each imaging modality was assessed using two measures. The first measure quantifies the overall fingerprinting accuracy across subjects and was calculated as the percentage of matrices which were successful in identifying an individual. While this measure is useful from a classification standpoint, we were also interested in the level of separation between matrices within versus between individuals. Therefore, in the second measure, we assessed the separability (*T*) of each modality. The separability of each matrix, T_*p*_, was defined to be

20 $${T}_{p}= \underset{\mathit{wit}h\mathit{in}-\mathit{subject}}{\mathrm{min}}\{{S}_{pq}\}- \underset{\mathit{between}-\mathit{subject}}{\mathrm{max}}\left\{{S}_{pq}\right\},$$

where the first term is constrained to *q* from the same subject as *p*, and the second term is constrained to q from all subjects other than p. The resulting values of *T* $\in$ [− 1 1], where a value of 1 indicates perfect similarity within a subject across sessions and no similarity to other subjects and, conversely, -1 indicates no similarity across runs within a subject.

### Statistical tests

Analysis of variance (ANOVA) was used to quantify the magnitude difference in ICC scores and the difference in the magnitude of the network similarity. Corresponding p-values were corrected for multiple comparison using Bonferroni correction. The Brain Connectivity Toolbox was used to calculate network measures^99^. All analyses were conducted in MATLAB 2017b.

## Supplementary Information

Supplementary Information.

## Data Availability

Data is available upon reasonable request to the corresponding authors and consequent approval by the US DEVCOM Army Research Laboratory and University of California, Santa Barbara.
