# Supplementary material for: Within-subject reproducibility varies in multi-modal, longitudinal brain networks
Source: Sci Rep. 2023 Apr 24;13:6699. doi: 10.1038/s41598-023-33441-3 (PMC10126005; doi:10.1038/s41598-023-33441-3)
Supplement: Supplementary file 1 — Supplementary Information. [file 41598_2023_33441_MOESM1_ESM.docx]

**Supplementary Information: Within-subject reproducibility varies in multi-modal, longitudinal brain networks**

Johan Nakuci^a,b^*, Nick Wasylyshyn^c,d^, Matthew Cieslak^e^, James C. Elliott^e^, Kanika Bansal^c,f^, Barry Giesbrecht^e,g^, Scott T. Grafton^e,g^, Jean M. Vettel^c,d,e^, Javier O. Garcia^c,d^, Sarah F. Muldoon^a,h^*

^a^ Neuroscience Program, University at Buffalo, SUNY, Buffalo, NY 14260, USA

^b^ School of Psychology, Georgia Institute of Technology, Atlanta, GA 14260, USA

^c^ U.S. CCDC Army Research Laboratory, Aberdeen Proving Ground, MD 21005, USA

^d^ Department of Bioengineering, University of Pennsylvania, Philadelphia, PA 19104, USA

^e^ Department of Psychological and Brain Sciences, University of California, Santa Barbara, CA 93106, USA

^f^ Department of Biomedical Engineering, Columbia University, New York, NY 10027, USA

^g^ Institute for Collaborative Biotechnologies, University of California, Santa Barbara, CA 93106, USA

^h^ Department of Mathematics and CDSE Program, University at Buffalo, SUNY, Buffalo, NY 14260 USA

^*^Corresponding Authors:

Johan Nakuci, [jnakuci3@gatech.edu](mailto:jnakuci3@gatech.edu)

Sarah F. Muldoon, [smuldoon@buffalo.edu](mailto:smuldoon@buffalo.edu)

**Figure S1**. **Intra-session half-split similarity.** For each subject and session, (A) fMRI and (B) EEG timeseries were split in half and functional connectivity was estimated on each half separately. Similarity in functional connectivity between the first and second halves of the session was then assessed using the Pearson correlation.

**Figure S2**. **QC-FC correlations.** Distribution of all edgewise QC-FC correlations after de-noising fMRI data.

**Figure S3**. **Distance-dependence of motion artifacts after de-noising.** Plots show the relation between the Euclidean distance between each pair of nodes and QC-FC correlation of the edges connecting the nodes. We observed no strong relationship between these variables.

**Figure S4**. **Graph measures** **intra-session half-split similarity.** The similarity between graph measures estimated from fMRI and EEG functional networks calculated from the first and second half of the timeseries for each subject and session. Similarity between graph measures between the first and second half of the session was estimated using the Pearson correlation.
